# Necroptosis: The Regulation Between EGFR and TNFR in Cancer

**DOI:** 10.3390/cells15141310

**Published:** 2026-07-22

**Authors:** Jin Gyeom Kim, Wook Jin

**Affiliations:** 1 Laboratory of Molecular Disease and Cell Regulation, Department of Biochemistry, School of Medicine, Gachon University, Incheon 21999, Republic of Korea; jingyeom916@naver.com; 2 Department of Health Sciences and Technology, GAIHST, Gachon University, Incheon 21999, Republic of Korea

**Keywords:** necroptosis, EGFR, TNFR, breast cancer

## Abstract

**Highlights:**

**What are the main findings?**

- This review summarizes recent advances in the molecular regulation of necroptosis and its multifaceted roles in cancer biology.
- Recent evidence identifies EGFR–TNFR1 signaling crosstalk and metabolic reprogramming as important modulators of necroptotic signaling.

**What are the implications of the main findings?**

- Understanding the mechanisms regulating necroptosis provides new opportunities to overcome therapeutic resistance in cancer.
- Integrating necroptosis-targeted strategies with existing anti-cancer therapies improves treatment efficacy and patient outcomes.

**Abstract:**

Most cancers are fatal and remain challenging to cure due to changes in tumorigenesis and therapeutic resistance. Despite significant advancements in cancer therapy, a substantial proportion of malignancies exhibit resistance to conventional therapies, driven primarily by cancer plasticity and the emergence of multidrug resistance. Recent cancer treatments include cytotoxic chemotherapy, molecular targeted therapy, and immune checkpoint inhibitors. A major hallmark of cancer cells is their ability to develop sophisticated evasion mechanisms that bypass programmed cell death when exposed to anti-cancer drugs. In addition, malignant cells evade the efficacy of anti-cancer drugs by altering cell proliferation, survival, and metastasis. To suppress oncogenic characteristics, necroptosis-based therapies have attracted substantial attention, as they can inhibit tumorigenesis and improve treatment outcomes across many cancer types. Furthermore, an increasing body of research focuses on suppressing tumorigenesis by targeting receptors that are overexpressed in cancer cells. In this review, we elucidate how tumorigenesis is inhibited by regulating the epidermal growth factor receptor (EGFR)–tumor necrosis factor receptor (TNFR) signaling pathway in necroptosis. By delineating the underlying mechanisms of these receptors, we propose that the induction of necroptosis via EGFR and TNFR represents an innovative paradigm for targeted therapy, offering a strategy to enhance clinical outcomes in treatment-resistant cancers.

## 1. Introduction

Cancer remains a leading cause of mortality worldwide. Specifically, it accounts for a large proportion of global deaths due to its complex causes and limited therapeutic options [1,2,3].

Cancer is driven by key biological hallmark processes, including sustained proliferative signaling, deregulated differentiation, angiogenesis, invasion, and metastasis. A critical hallmark of cancer is the evasion of programmed cell death, which, together with genetic instability and metabolic reprogramming, drives tumorigenesis and treatment resistance [4,5,6]. Therefore, overcoming cell death evasion represents a key objective of modern cancer therapy.

Over the past decades, significant advances have been made in cancer therapy, including surgery, radiotherapy, antibody–drug conjugates (ADCs), immune checkpoint inhibitors (ICIs), cell death-based treatments, and gene-targeted therapies [6,7,8,9,10]. Within this landscape, the regulation of necroptosis is associated with cancer initiation, progression, metastasis, and drug resistance. While the activation of necroptosis suppresses malignant cell growth and proliferation, cancer cells evade or repair cell death signaling pathways to promote cell growth, proliferation, and metastasis [11,12,13]. To overcome such resistance, recent studies have explored combination therapies, ADC-based strategies, ICI-mediated regulation of target genes, and clinical applications of cell death induction [14,15,16].

This review focuses on necroptosis in cancer. Based on these findings, we suggest a strategy to maximize therapeutic efficacy in treatment-resistant malignancies. Necroptosis, a regulated and pro-inflammatory form of lytic cell death mediated by receptor-interacting protein kinases 1 and 3 (RIPK1/RIPK3) and the mixed lineage kinase domain-like protein (MLKL), has emerged as a promising therapeutic target, particularly in cancers such as colorectal cancer and leukemia that develop resistance to cell death-inducing therapies. Unlike necrosis, necroptosis is regulated through the RIPK1–RIPK3–MLKL signaling axis. The activation of these signaling pathways promotes MLKL oligomerization and plasma membrane permeabilization, thereby facilitating a necrotic morphology accompanied by inflammatory signaling [17,18,19].

In contrast to necroptosis, necrosis has long been viewed as an accidental, passive, and unregulated process, referred to as accidental cell death (ACD). Morphologically, ACD is characterized by cell swelling, organelle distention, plasma membrane rupture, and uncontrolled leakage of intracellular constituents into the extracellular environment. This loss of membrane integrity leads to the release of damage-associated molecular patterns (DAMPs)–such as high mobility group box 1 (HMGB1), ATP, nucleic acids, and heat shock proteins–which drive a robust inflammatory response and exacerbate tissue damage [20,21,22]. Unlike necroptosis and necrosis, apoptosis–a regulated form of programmed cell death–is essential for homeostasis and immune regulation. Morphologically, it is characterized by cytoplasmic condensation, membrane blebbing, chromatin fragmentation, and the formation of apoptotic bodies, which are subsequently cleared by efferocytosis, thereby maintaining immunological quiescence [23,24].

In this review, we delineate the molecular mechanisms of necroptosis, elucidate its functional role in cancer, and outline novel approaches to improve therapeutic outcomes.

## 2. Cell Death

Cell death is broadly categorized into regulated cell death (RCD) and ACD. RCD is a genetically regulated process in which cells undergo programmed death in response to specific physiological or pathological stimuli. In contrast, ACD is characterized by the instantaneous, physical destruction of cells [20].

### 2.1. Apoptosis

**Figure 1:**
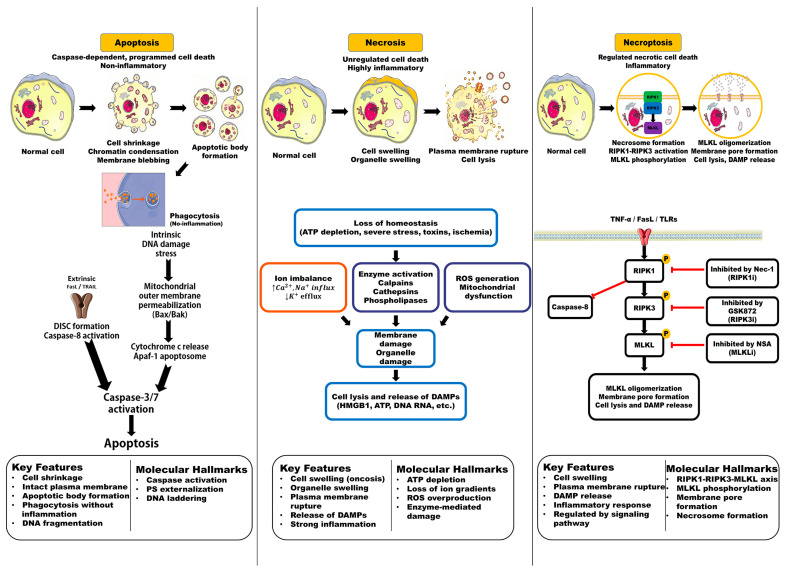
**Mechanism of Apoptosis, Necrosis, and Necroptosis.** Cell death can be broadly classified into three major forms (apoptosis, necrosis, and necroptosis), which differ in their degree of regulation and their ability to elicit inflammation. Apoptosis is a caspase-dependent, programmed, non-inflammatory form of cell death that is mediated by extrinsic or intrinsic pathways. Necrosis is unregulated, highly inflammatory, and triggered by a loss of homeostasis (ATP depletion, severe stress, ischemia). Necroptosis shows a regulated form of necrotic, inflammatory cell death initiated by ligands like TNF-α, FasL, or TLRs. The figure was drafted through Servier Medical Art, revised through the free version of ChatGPT (GPT-5.5), and then completed through Servier Medical Art.

- **Physiological Features:** Apoptosis represents the paradigm of RCD, playing an indispensable role in developmental morphogenesis and the maintenance of immunological tolerance. Morphologically characterized by chromatin fragmentation, cytoplasmic condensation, and the formation of distinct apoptotic bodies, apoptotic cells are swiftly cleared via efferocytosis to preserve immunological quiescence within the tissue microenvironment (Table 1 and Figure 1) [25].

**Table 1:** **Comparison of necroptosis, necrosis, and apoptosis in cell death** [18,19,26,27,28,29,30,31,32,33]. The symbols represent the relative level of reported papers rather than quantitative expression levels. “+/−” indicates controversial or context-dependent findings, “+” indicates limited evidence, “++” indicates multiple independent reports, and “+++” indicates extensive and consistently demonstrated evidence.

	Characteristic	Necroptosis	Necrosis	Apoptosis
Contained proteins	RIPK1	+++	−	+
	RIPK3	+	−	−
	MLKL	+	−	−
	Caspase-3	−	−	+
	Membrane perforation	+++	+++	−
Morphological characteristics	Membrane blebbing	−	−	+
	DNA fragmentation	+	+/−	+++
	Cell lysis	+	+	−
	Cell swelling	+	+	−
	Inflammation	+	+	+/−
Receptors	EGFR	+	+/−	+++
	TNFR	+++	+/−	+++
Cancer types	Breast cancer	+++	++	++
	Lung cancer	+++	++	++
	Colorectal cancer	++	+++	++
	Pancreatic cancer	++	+++	+++
	Melanoma	+	++	+++
	Liver cancer	+	+++	+++

- **Signaling Pathways (Intrinsic and Extrinsic):** The execution of apoptosis occurs through two primary interconnected pathways. Intracellular stress signals trigger the intrinsic (mitochondrial) pathway and are tightly regulated by the B-cell lymphoma 2 (BCL-2) family of proteins, which regulate mitochondrial outer membrane permeabilization. In contrast, the extrinsic pathway is initiated by the engagement of cell-surface death receptors (e.g., Fas Cell Surface Death Receptor (Fas), TNF-related apoptosis-inducing ligand (TRAIL), and Tumor Necrosis Factor Receptor 1 (TNFR1) by their respective ligands, assembling the death-inducing signaling complex to activate initiator caspase-8. Both pathways ultimately converge on the activation of executioner caspases, specifically caspase-3 and caspase-7, to systematically dismantle structural and regulatory proteins within the cell (Table 1) [25,34,35,36,37].
- **Pharmacological Regulation and Therapies:** In the context of cancer therapy, pharmacological strategies targeting apoptotic resistance should be considered within the broader framework of TNFR1-dependent cell death regulation [18,38,39]. BH3 mimetics, such as venetoclax, restore apoptotic sensitivity by inhibiting anti-apoptotic BCL-2 family proteins; however, their efficacy is frequently limited by compensatory survival pathways, including MCL-1-mediated resistance [40,41]. Also, SMAC mimetics, including LCL161 and birinapant, enhance death receptor-mediated apoptotic signaling by targeting inhibitors of apoptosis proteins (IAPs) [42,43,44,45,46,47,48,49,50,51]. These agents induce cIAP1/2 degradation, destabilize TNFR1-associated Complex I, promote RIPK1 deubiquitination, and facilitate formation of the ripoptosome, a RIPK1-containing intracellular death platform that can determine apoptotic or necroptotic outcomes depending on caspase-8 activity and cellular context [42,43,44,45,46,52]. Importantly, EGFR-mediated attenuation of TNFR1-dependent death signaling and SMAC mimetic-induced promotion of RIPK1-dependent death complex formation represent opposing regulatory mechanisms that converge at the level of TNFR1-RIPK1 signaling [45,53]. This convergence suggests that the EGFR–TNFR1–RIPK1 axis represents a potential therapeutic vulnerability. Within this signaling network, RIPK1 serves as a pivotal molecular checkpoint that determines the balance between cell survival and apoptosis or necroptosis [18,26,54,55,56]. Future therapeutic strategies should consider the molecular context of tumors, including EGFR activation status, TNFR1 expression, and RIPK1 regulatory state, to identify tumor subsets most likely to benefit from combined EGFR inhibition and TNFR1-dependent death pathway activation (Table 2).

### 2.2. Necrosis

- **Morphological Collapse in ACD:** Necrosis has historically been defined as a passive, uncontrolled form of ACD precipitated by extreme environmental stresses, such as severe hypoxia, hyperthermia, and mechanical trauma [57,58,59]. Severe bioenergetic catastrophe leads to the failure of ATP-dependent ion pumps, provoking an unregulated influx of calcium (Ca^2+^) and sodium (Na^+^). Cytosolic calcium overload indiscriminately activates deleterious hydrolytic enzymes—including calpains, cathepsins, and phospholipases—permanently degrading the cellular proteome and lipid bilayers [60,61,62,63]. Concurrently, intracellular sodium accumulation drives an osmotic imbalance, inducing massive water influx, cellular and organelle swelling (oncosis), and culminating in catastrophic plasma membrane rupture (Table 1 and Figure 1) [64,65,66].
- **Inflammatory Microenvironment and DAMP Release:** The complete loss of membrane integrity triggers the rapid, indiscriminate leakage of intracellular components and DAMPs—such as HMGB1, ATP, genomic DNA, and heat shock proteins—into the extracellular milieu [67,68,69,70,71]. These DAMPs act as potent danger signals that instigate a robust innate immune response, driving chronic inflammation within the TME. In the context of malignancy, this necrosis-induced chronic inflammation can act as a double-edged sword, paradoxically fueling tumor progression by stimulating cell proliferation, remodeling the extracellular matrix, and promoting angiogenesis and metastasis (Table 1) [72,73,74].
- **Expansion into Regulated Necrosis (RN):** In contrast to accidental necrosis, recent paradigms recognize several RN pathways that operate under precise genetic programs and signaling axes [18,19]. Key modalities include necroptosis, driven by the RIPK1–RIPK3–MLKL axis leading to regulated osmotic lysis; ferroptosis, characterized by glutathione peroxidase 4 (GPX4) inhibition and iron-dependent lipid peroxidation; pyroptosis, involving inflammasome activation and Gasdermin D pore formation; and parthanatos, mediated by poly(ADP-ribose) polymerase-1 (PARP-1) hyperactivation resulting in nicotinamide adenine dinucleotide (NAD+) depletion and apoptosis-inducing factor (AIF) nuclear translocation [19,75,76,77].
- **Necrosis Resistance and Therapeutic Outlook:** To withstand plasma membrane damage and mechanical stress, advanced cancer cells deploy adaptive survival strategies. These include upregulating the Endosomal Sorting Complex Required for Transport III (ESCRT-III) to dynamically repair damaged membranes and delay osmotic lysis, as well as enhancing antioxidant buffering capacities and rewiring mitochondrial metabolic pathways [78,79,80,81]. Consequently, elucidating these multifaceted resistance mechanisms is pivotal to counteract cell death evasion and optimize tumor-killing efficacy in clinical settings.

To circumvent the therapeutic limitations imposed by apoptotic evasion and avoid the detrimental inflammatory consequences of accidental necrosis, recent oncological research has increasingly focused on necroptosis. As a form of regulated necrotic cell death, necroptosis shares the morphological hallmarks of necrosis but operates under a highly orchestrated, genetically programmed signaling cascade, offering a promising alternative paradigm to overcome treatment resistance in cancer.

### 2.3. Necroptosis

- **Physiological Features and Therapeutic Rationale:** To circumvent the therapeutic limitations imposed by apoptotic evasion while avoiding the detrimental inflammatory consequences associated with accidental necrosis, contemporary oncological research has increasingly focused on necroptosis. As a representative modality of RN, necroptosis combines the morphological hallmarks of necrotic cell death—including organelle swelling, bioenergetic collapse, and terminal plasma membrane rupture—with a tightly regulated signaling cascade. This dual nature positions necroptosis as a promising alternative strategy for eliminating refractory tumors that exhibit intrinsic resistance to conventional apoptosis-inducing therapies, while potentially enabling a more controlled modulation of downstream immune responses (Table 1 and Figure 1) [82,83,84,85,86,87].
- **Signaling Pathways (The TNFR1-Necrosome Axis and Organelle Dysfunction):** The molecular initiation of necroptosis is primarily mediated by TNFR1, a pleiotropic death receptor implicated in inflammatory signaling, tumor progression, and metastatic dissemination across multiple malignancies. Upon TNF-α ligation, TNFR1 recruits tumor necrosis factor receptor type 1-associated death domain (TRADD), TNF receptor-associated factor 2 (TRAF2), cIAPs, and the linear ubiquitin chain assembly complex (LUBAC) to assemble membrane-associated Complex I. The signaling fate of Complex I is critically regulated by RIPK1’s ubiquitination status. Under pro-survival conditions, cIAP1/2-mediated K63-linked polyubiquitination of RIPK1 stabilizes Complex I and promotes downstream nuclear factor kappa-light-chain-enhancer of activated B cells (NF-κB) signaling. In contrast, commitment to cell death is initiated when deubiquitinases, particularly cylindromatosis (CYLD), dismantle these ubiquitin scaffolds, allowing RIPK1 to dissociate from the plasma membrane and translocate into the cytosol (Figure 1) [38,88,89,90].

Within the cytosolic compartment, RIPK1 associates with Fas-associated protein with death domain (FADD) and pro-caspase-8 to form Complex II. The activation threshold of necroptosis is critically regulated at this checkpoint; functional caspase-8 cleaves and inactivates RIPK1 and RIPK3, thereby diverting the cell toward apoptosis. In contrast, under conditions of caspase-8 deficiency or pharmacological inhibition, RIPK1 interacts with RIPK3 through their RIP homotypic interaction motif (RHIM) domains. This interaction promotes reciprocal phosphorylation events within the RIPK1–RIPK3 axis, leading to the recruitment and phosphorylation of MLKL and the subsequent assembly of the necrosome [18,19,39,91]. The conformational remodeling and oligomerization of phosphorylated MLKL mediate the terminal execution phase of necroptosis. Activated MLKL translocates to the plasma membrane, where it interacts with phosphoinositides and disrupts membrane integrity, thereby perturbing ion homeostasis and promoting osmotic lysis. Necroptosis is additionally associated with mitochondrial dysfunction, reactive oxygen species (ROS) accumulation, and bioenergetic collapse, which facilitate the release of mitochondrial damage-associated molecular patterns, including mitochondrial DNA (mtDNA). Cytosolic mtDNA subsequently engages the cyclic GMP-AMP synthase-stimulator of interferon genes (cGAS–STING) innate immune signaling pathway, thereby amplifying local inflammatory responses and immunogenic signaling (Figure 1) [27,28,82,92,93,94,95,96].

- **Pharmacological Regulation and Cancer Therapies:** In oncology, therapeutic induction of necroptosis has emerged as a promising strategy to overcome apoptosis resistance in refractory malignancies. Pharmacological activation of necroptosis commonly involves the combined use of SMAC mimetics to deplete cIAPs together with pan-caspase inhibitors (e.g., z-VAD-fmk) under TNF-α stimulation, thereby facilitating necrosome assembly and necroptotic signaling [39,45,97,98]. Emerging small-molecule sensitizers and epigenetic modifiers, including DNA methyltransferase (DNMT) inhibitors, aim to restore RIPK3 or MLKL expression in tumors where these components are epigenetically silenced, thereby re-sensitizing subsets of TNBC and melanoma to immunogenic cell death [99,100]. Furthermore, combining necroptosis-inducing therapies with immune checkpoint blockade (e.g., anti-PD-1 antibodies) exploits necroptosis-associated DAMP release to convert immunologically cold tumors into hot, cytotoxic T lymphocyte (CTL)-driven responses (Table 2) [86,101,102].

**Table 2:** **Pharmacological regulation of regulated cell death pathways and therapeutic implications in cancer** [13,18,19,26,39,53,103,104,105,106,107,108,109].

Cell Death Modality	Therapeutic Strategy/Agent	Molecular Target	Mechanism of Action	Cancer Therapeutic Implication
Necroptosis induction	TNF-α + z-VAD-fmk	TNFR1–RIPK1–RIPK3–MLKL axis	Caspase-8 inhibition prevents apoptosis and promotes RIPK1/RIPK3 necrosome assembly and MLKL activation	Induces necroptosis in apoptosis-resistant cancer cells
	SMAC mimetics (Birinapant, LCL161)	cIAP1/2 degradation	Induces cIAP loss, RIPK1 deubiquitination, destabilization of TNFR1 Complex I, and ripoptosome formation	Sensitizes tumor cells to TNFR1-dependent apoptosis/necroptosis
	DNMT inhibitors	RIPK3 promoter methylation	Reactivation of epigenetically silenced RIPK3 or MLKL expression	Restores necroptotic sensitivity in RIPK3-deficient cancers
Necroptosis inhibition	Necrostatin-1s	RIPK1 kinase	Selectively inhibits RIPK1 kinase activity while preserving scaffold function	Prevents pathological necroptosis and DAMP-mediated inflammation
	GSK872	RIPK3 kinase	Blocks RIPK3 activation and prevents necrosome signaling	Suppresses excessive RIPK3-dependent tissue injury
	Necrosulfonamide	MLKL	Prevents MLKL oligomerization and membrane translocation	Inhibits terminal execution of necroptosis
Apoptosis induction	BH3 mimetics (Venetoclax)	BCL-2 family proteins	Inhibits anti-apoptotic BCL-2 proteins and restores mitochondrial apoptosis	Effective in hematologic malignancies; limited by MCL-1-mediated resistance
	TRAIL/FasL	DR4/DR5/Fas	Activates DISC formation and caspase-8-dependent apoptosis	Exploits death receptor-mediated apoptosis
	Cisplatin	DNA damage response	Induces DNA damage and mitochondrial apoptotic signaling	Widely used cytotoxic chemotherapy
	SMAC mimetics	IAP proteins (cIAP1/2, XIAP)	Removes apoptosis inhibitors and promotes caspase activation	Enhances death receptor-mediated apoptotic sensitivity
Necrosis	Chemotherapy-induced oxidative stress (e.g., high-dose anthracyclines, alkylating agents)	ROS, mitochondria, ATP metabolism	Excessive ROS production and mitochondrial damage impair energy metabolism, causing irreversible cellular injury and necrotic death	Contributes to cytotoxic efficacy of anticancer drugs; necrosis-associated inflammation may influence tumor microenvironment
	ROS-generating compounds (e.g., pro-oxidant agents)	Mitochondrial ROS, redox homeostasis	Induction of oxidative stress overwhelms antioxidant capacity, resulting in membrane damage and necrotic cell death	Potential strategy to target tumors with elevated oxidative stress dependency
	ATP depletion-based metabolic targeting	Glycolysis, mitochondrial respiration, AMPK pathway	Severe metabolic stress reduces ATP production, preventing maintenance of ion gradients and causing necrotic membrane rupture	Exploits metabolic dependence of cancer cells; potential combination strategy with metabolic inhibitors
	Necrosis-associated immunotherapy strategies	DAMPs (HMGB1, ATP, mitochondrial DNA), immune activation	Necrotic cell death releases immunostimulatory molecules that activate dendritic cells and antitumor immunity	May enhance immune checkpoint blockade responses by increasing tumor immunogenicity
	Inhibition of necrosis-associated inflammation	TLRs, NF-κB, inflammasome signaling	Blocking inflammatory signaling triggered by necrotic DAMP release reduces tumor-promoting inflammation	May prevent chronic inflammation-driven tumor progression after therapy-induced necrosis

- **Cytoprotective Strategies in Pathological Necroptosis:** Excessive or dysregulated necroptosis has been implicated in the pathogenesis of acute ischemic stroke, myocardial infarction, and systemic inflammatory response syndrome (SIRS) [26,110,111]. To limit pathological tissue damage, cytoprotective interventions target multiple upstream and downstream nodes within the necroptotic pathway. Small-molecule RIPK1 inhibitors, particularly Necrostatin-1s (Nec-1s), provide neuroprotective and cardioprotective effects by selectively inhibiting RIPK1 kinase activity while preserving its scaffolding functions [26,110,112]. Additionally, selective RIPK3 inhibitors (e.g., GSK872) and MLKL inhibitors (e.g., Necrosulfonamide/NSA) are under investigation for their ability to suppress MLKL activation and membrane translocation, thereby attenuating necroptotic tissue injury and subsequently reducing chronic DAMP-mediated sterile inflammation (Table 2) [19,103,104,113].
- **Mechanisms of Necroptotic Evasion and Tumor-Promoting Effects of Necroptosis:** Advanced tumor cells acquire multiple strategies to evade necroptosis, thereby surviving cytotoxic stress and reducing their sensitivity to necroptosis-inducing therapies. One of the best-characterized mechanisms of necroptotic evasion is the epigenetic silencing of RIPK3 via promoter hypermethylation, resulting in impaired necroptotic signaling and defective execution of necroptotic cell death [13,39]. Restoration of RIPK3 expression re-sensitizes colorectal cancer and breast cancer cell lines to necroptosis, demonstrating that epigenetic repression of RIPK3 is functionally reversible [13]. Similar epigenetic inactivation of RIPK3 has also been reported in malignant mesothelioma and non-small cell lung cancer, where promoter methylation suppresses necroptotic signaling, contributes to chemoresistance, and can be reversed by pharmacological demethylation or ectopic RIPK3 re-expression [99,114]. Taken together, these studies indicate that epigenetic repression of RIPK3 represents a recurrent mechanism by which the RIPK1–RIPK3–MLKL pathway is functionally inactivated, thereby enabling tumor cells to evade necroptosis and acquire resistance to cytotoxic therapies.

Beyond RIPK3 epigenetic silencing, additional regulatory mechanisms influence the threshold for necroptotic activation. Caspase-8 suppresses RIPK3-dependent necroptosis by restricting RIPK1–RIPK3 necrosome formation, whereas cFLIP-containing caspase-8 complexes further inhibit necroptotic activation and regulate cell fate decisions [115,116]. These checkpoints collectively determine whether TNFR1 signaling promotes cell survival or necroptotic cell death under specific cellular contexts.

Furthermore, genetic alterations affecting core necroptotic mediators represent an additional mechanism contributing to functional impairment of the necroptotic machinery in cancer. Somatic mutations in RIPK1, RIPK3, and MLKL have been identified in human cancers, including gastric and colorectal cancers, and are predicted to disrupt kinase activity, protein stability, or protein–protein interactions required for necrosome assembly. Among these alterations, MLKL mutations affecting conserved residues within the pseudokinase domain, including F398I and L291P identified in human gastric cancer, have been suggested to interfere with the conformational switch required for MLKL activation, thereby impairing MLKL oligomerization, membrane translocation, and downstream plasma membrane disruption during necroptosis [117]. Also, functional studies have demonstrated that RIPK1 and RIPK3 contribute differently depending on the type of cell death stimulus. TNF-α-induced necroptosis requires coordinated RIPK1/RIPK3 activation, whereas chemotherapeutic agent-induced cell death exhibits differential dependency on these mediators, indicating that RIPK1 and RIPK3 function in a stimulus-dependent manner and influence cellular responses to different therapeutic stresses [29]. In addition, genetic mutation in RIPK3 has been associated with an increased risk of non-Hodgkin lymphoma, supporting the concept that altered RIPK3 contributes to impaired necroptotic signaling during tumorigenesis [13,29,99,118].

Notably, although controlled necroptotic activation can enhance anti-tumor immunity, persistent or dysregulated necroptosis paradoxically promotes tumor progression by driving inflammatory remodeling of the tumor microenvironment [12,102,119]. RIPK3-dependent inflammatory signaling stimulates the production of cytokines and chemokines that recruit immunosuppressive myeloid populations, including tumor-associated macrophages and myeloid-derived suppressor cells, thereby reinforcing an immunosuppressive tumor microenvironment [119,120,121].

Together, these studies underscore that the biological outcome of necroptosis is governed not only by the activation of the necroptotic machinery itself but also by the balance between upstream survival and death-regulatory signals within the tumor context. Therefore, elucidating how EGFR-mediated survival signaling interfaces with TNFR1-dependent death pathways to control RIPK1-mediated cell fate decisions will be critical for developing rational and context-specific necroptosis-based therapeutic strategies.

## 3. Necroptosis-Targeting Therapeutics

Necroptosis is a highly regulated form of programmed necrosis, fundamentally governed by the assembly of the necrosome, a signaling complex primarily composed of RIPK1, RIPK3, and the executioner protein MLKL [18,19,39,91]. This process is dynamically regulated by ubiquitination-dependent signaling, caspase-8 activity, and the balance of pro-survival versus pro-death inflammatory networks within the tissue microenvironment [103,115,122,123]. Recent advances in necroptosis-targeting therapeutics have positioned this pathway as a promising strategy to overcome apoptosis resistance in immunologically cold tumors and to reshape the tumor microenvironment toward a more pro-inflammatory, immune-permissive state. Moreover, accumulating evidence suggests that pharmacological modulation of necroptotic signaling holds therapeutic potential beyond oncology, including ischemia–reperfusion injuries, neurodegenerative disorders, and SIRS, where dysregulated necroinflammatory signaling contributes to tissue destruction and organ dysfunction [97,102,110,111,124,125].

Collectively, therapeutic manipulation of the RIPK1–RIPK3–MLKL signaling pathway is increasingly being recognized not merely as a mechanism for inducing cell death but as a broader strategy for reprogramming pathological inflammatory states across diverse disease contexts.

### 3.1. Death Receptor-Mediated Induction and IAP Antagonism

The canonical induction of necroptosis is predominantly initiated through TNFR1-dependent signaling. Upon binding of TNF-α to TNFR1, TNFR1 recruits multiple adaptor proteins to assemble the membrane-associated complex I, a signaling platform composed of TRADD, RIPK1, TRAF2/5, cIAP1/2, and LUBAC [38,126,127]. Under homeostatic conditions, cIAP1 and cIAP2 function as critical E3 ubiquitin ligases that conjugate K63-linked polyubiquitin chains onto RIPK1, thereby stabilizing pro-survival signaling platforms and suppressing aberrant cell death activation. These RIPK1-associated ubiquitin scaffolds facilitate the recruitment of TAK1-binding protein 2/3 (TAB2/3)–transforming growth factor-beta-activated kinase-1 (TAK1) and IκB kinase (IKK) signaling complexes, resulting in canonical NF-κB activation. Subsequent IKK-mediated phosphorylation and proteasomal degradation of I-kappa-B-alpha (IκBα) permit NF-κB nuclear translocation and the transcriptional induction of multiple pro-survival mediators, including cellular FLICE-inhibitory protein (cFLIP), cIAP2, XIAP, and anti-apoptotic BCL-2 family proteins [122,128]. Beyond functioning as molecular scaffolds, RIPK1-associated ubiquitin chains serve as critical inhibitory checkpoints that restrain RIPK1 kinase activation and prevent its dissociation from membrane-associated Complex I into cytosolic death-inducing complexes. In this context, TAK1- and IKK-dependent inhibitory phosphorylation cooperates with ubiquitin-mediated signaling to maintain RIPK1 in a non-cytotoxic state. Consequently, cIAP1/2-mediated ubiquitination suppresses the formation of RIPK1-dependent cytosolic Complex II and limits pathological activation of necroptotic signaling pathways [129]. Necroptosis is triggered when this ubiquitin-dependent checkpoint collapses, particularly following pharmacological antagonism or depletion of cIAP1/2. The loss of cIAP1/2 destabilizes TNFR1 Complex I and facilitates RIPK1 deubiquitination by CYLD and related ubiquitin-editing enzymes, thereby permitting RIPK1 kinase activation and its subsequent dissociation from membrane-associated signaling complexes. In environments where caspase-8 activity is concomitantly compromised, this kinase-active RIPK1 initiates the downstream necroptotic cascade [45,130]. In multiple IAP-amplified malignancies, aberrant overexpression of cIAP1/2 establishes a potent survival checkpoint that simultaneously suppresses TNF-induced apoptosis and necroptosis while sustaining constitutive NF-κB signaling. In addition to conferring resistance to inflammatory cytotoxicity, persistent cIAP-dependent signaling promotes oncogenic fitness by inducing mitogenic and anti-apoptotic transcriptional programs, including Cyclin D1 and anti-apoptotic BCL-2 family members. This dual capacity to suppress programmed cell death while reinforcing proliferative signaling highlights cIAP1/2 as a central determinant of tumor survival and a compelling therapeutic target for necroptosis-sensitizing strategies in apoptosis-refractory cancers [105,106,122,131].

### 3.2. The Pivotal Role of CYLD in RIPK1 Activation

The transition from ubiquitin-restrained TNFR1 signaling to RIPK1-dependent necroptosis critically depends on the coordinated dismantling of inhibitory ubiquitin checkpoints. Central to this process is the deubiquitinase CYLD, which is recruited to the TNFR1 receptor signaling complex via the adaptor protein spermatogenesis-associated protein 2 (SPATA2); this adaptor directly bridges CYLD to the HOIL-1-interacting protein (HOIP) subunit of the LUBAC. Within this signaling platform, CYLD catalyzes the selective removal of K63-linked and M1-linked polyubiquitin chains from RIPK1 and LUBAC-associated components, thereby destabilizing the ubiquitin scaffolds that sustain TAK1- and IKK-mediated pro-survival signaling. This ubiquitin-editing process not only attenuates NF-κB activation but also relieves RIPK1 of inhibitory constraints that normally suppress its kinase activity [132,133,134]. In particular, CYLD-dependent deubiquitination cooperates with the collapse of inhibitory signaling mediated by the IKK complex and the TAK1–p38–MK2 axis, thereby licensing RIPK1 autophosphorylation and cytotoxic activation [135,136]. Concurrently, kinase-active RIPK1 undergoes autophosphorylation at Ser166, a widely recognized marker of RIPK1 kinase activation that promotes its transition into cytosolic death-inducing signaling complexes. Under conditions in which caspase-8-mediated suppression is impaired, RIPK1 engages RIPK3 through RHIM-dependent interactions, resulting in the assembly of the necrosome. This amyloid-like supramolecular signaling platform drives RIPK3 phosphorylation and downstream MLKL activation [18,19,39].

Consequently, CYLD-mediated ubiquitin editing functions as a decisive molecular switch, converting RIPK1 from a scaffold-associated survival regulator into a catalytic necroptotic signaling hub, thereby establishing CYLD as a central rheostat governing inflammatory cell fate determination.

### 3.3. TLR and Interferon-Driven Necroptosis

Necroptosis can also be initiated independently of canonical death receptor signaling through innate immune sensing pathways, particularly Toll-like receptor 3/4 (TLR3/TLR4) and the TIR domain-containing adaptor inducing interferon-β (TRIF), as well as type I interferon (IFN) signaling. In specific cellular contexts, TLR3 and TLR4 activation by pathogen-associated molecular patterns–including viral double-stranded RNA and lipopolysaccharide–can induce necroptotic signaling through the RHIM-containing adaptor TRIF, which recruits RIPK3 independently of canonical death receptor signaling. Unlike TNFR1 signaling, TRIF contains an RHIM that enables direct engagement of RIPK3. Although this pathway bypasses the requirement for membrane-associated TNFR1 Complex I, TRIF-mediated necroptosis can involve RIPK1 kinase activity to facilitate or amplify necrosome assembly in a context-dependent manner. This alternative mode of necroptotic activation highlights the integration of necroptosis within innate immune surveillance and antiviral host defense pathways [27,103,137,138]. In parallel with TLR-mediated surveillance, type I interferon signaling further enhances necroptotic susceptibility by upregulating Z-DNA-binding protein 1 (ZBP1), also known as the DNA-dependent activator of interferon regulatory factors (DAI). ZBP1 functions as a cytosolic nucleic acid sensor that recognizes Z-form nucleic acid conformations generated during viral replication and cellular stress responses. Similar to TRIF, ZBP1 contains RHIM domains that directly engage RIPK3, thereby promoting RIPK3 activation independently of upstream death receptor signaling. Interestingly, whereas RIPK1 functions as a pro-necroptotic initiator in canonical TNFR1 pathways, it can also serve as a scaffold-dependent suppressor that restrains aberrant ZBP1–RIPK3 interactions under homeostatic conditions. ZBP1-driven necroptosis has emerged as a critical component of antiviral immunity during infections caused by influenza A virus, herpesviruses, vaccinia virus, and other cytopathic pathogens. However, dysregulated ZBP1–RIPK3 signaling has also been implicated in pathological inflammation, cytokine amplification, and tissue injury in sterile inflammatory disorders and severe viral syndromes [55,139,140].

Despite substantial diversity in their upstream sensing mechanisms, many necroptotic pathways ultimately converge on RIPK3-mediated phosphorylation and activation of MLKL when caspase-8-dependent suppressive signaling is impaired. Under physiological conditions, caspase-8 functions not only as a central initiator of extrinsic apoptosis but also as a critical suppressor of necroptosis through proteolytic dismantling of RIPK1–RIPK3-associated signaling complexes. Consequently, genetic deficiency, viral antagonism, or pharmacological inhibition of caspase-8 can shift inflammatory signaling networks toward necroptotic execution [103,115,141]. Collectively, these observations position necroptosis as a central inflammatory effector program that integrates death receptor signaling, innate immune sensing, and antiviral defense pathways into a convergent RIPK3–MLKL-mediated execution machinery.

The translational potential of these pathways has driven extensive research into the therapeutic targeting of specific nodes within the RIPK1–RIPK3–MLKL signaling axis. Nec-1, the prototypical RIPK1 inhibitor, suppresses RIPK1 kinase activity, thereby preventing the initiation of necrosome assembly. Preclinical studies have demonstrated the protective effects of Nec-1 in models of ischemia–reperfusion injury, neurodegeneration, inflammatory disorders, and sepsis-associated tissue injury. The therapeutic implications of RIPK1 inhibition in oncology remain highly context-dependent, as suppression of necroptosis simultaneously attenuates pathological inflammation while limiting immunogenic tumor cell death and anti-tumor immune activation [18,19,39]. To address these limitations, next-generation RIPK1 inhibitors with improved pharmacokinetic properties, including GSK2982772 and related compounds, have advanced into clinical evaluation for the treatment of chronic inflammatory diseases. At the level of RIPK3, GSK872 selectively inhibits RIPK3 kinase activity and suppresses downstream MLKL phosphorylation.

Nevertheless, prolonged or high-dose RIPK3 inhibition has been associated with paradoxical scaffold-dependent apoptotic signaling, reflecting the conformational plasticity of RIPK3 and the broader complexity of therapeutically targeting regulated cell death pathways. Downstream inhibition of terminal necroptotic execution can also be achieved using NSA, which covalently binds the Cys86 residue of human MLKL, preventing MLKL oligomerization and plasma membrane translocation. However, NSA exhibits marked species specificity and does not effectively inhibit murine MLKL, thereby complicating translational interpretation in preclinical animal models [19,26,28,104,113]. Despite these advances, major therapeutic challenges remain unresolved. For instance, inhibition of necroptotic signaling can result in compensatory engagement of alternative cell death programs, including apoptosis, pyroptosis, and integrated PANoptotic signaling networks, thereby complicating durable therapeutic intervention [103,115,142,143].

In contrast to pharmacological inhibition of necroptotic signaling, therapeutic activation of necroptosis has emerged as a potential strategy to exploit the immunogenic properties of this cell death pathway. Necroptotic tumor cells can stimulate protective anti-tumor immunity through coordinated immunogenic signaling, in which DAMP release promotes dendritic cell maturation, cross-priming of cytotoxic T cells, and tumor antigen-specific adaptive immune responses, whereas RIPK3-dependent type I interferon signaling amplifies these immunogenic effects and contributes to durable anti-tumor immunity [86,144]. However, recent studies indicate that necroptosis can also exert pro-tumorigenic effects in a context-dependent manner. Tumor cell-induced endothelial necroptosis via death receptor 6 (DR6) signaling disrupts endothelial barrier function, thereby facilitating tumor progression [12]. Furthermore, in pancreatic cancer, RIPK1–RIPK3 necrosome activation drives the establishment of an immunosuppressive tumor microenvironment through CXCL1-mediated recruitment of myeloid cells and Mincle-dependent inflammatory signaling, thereby promoting tumor persistence and progression [119]. Taken together, the dual roles of necroptosis underscore the need for therapeutic strategies that precisely modulate necroptotic signaling to maximize anti-tumor immunogenicity while minimizing pro-tumorigenic inflammation.

## 4. Necroptosis: The Regulation of the Interaction Between EGFR and TNFR

Cell death has long been the primary focus of oncology; the emergence of treatment resistance necessitates a deeper understanding of alternative RCD pathways, particularly necroptosis, and their regulation by oncogenic receptors such as EGFR and TNFR [4,6,20,39,53].

EGFR primarily promotes tumorigenesis by activating the JAK2/STAT3, PI3K/AKT, and MAPK/ERK signaling pathways [145,146,147,148]. Beyond these canonical functions, recent studies indicate that EGFR directly modulates TNFR1-mediated cell death signaling through interaction with TNFR1 and phosphorylates the TNFR1 death domain at Tyr360 and Tyr401, thereby limiting RIPK1 to the TNFR1 signaling complex and suppressing TNF-α-induced necroptosis [53]. This finding provides direct mechanistic evidence that EGFR negatively regulates TNFR1-dependent necroptotic signaling by phosphorylating TNFR1. In addition to its direct regulation of TNFR1 signaling, EGFR activates downstream kinase networks that can contribute to the regulation of necroptotic susceptibility. Among these signaling mediators, c-Src is a well-established downstream effector of EGFR activation and contributes to EGFR-driven oncogenic signaling by regulating tumor cell proliferation, survival, and progression across multiple cancer types, including colorectal cancer, head and neck squamous cell carcinoma, glioblastoma, and non-small cell lung cancer [149,150,151,152,153,154].

EGFR and c-Src function cooperatively, with activated c-Src phosphorylating EGFR at Tyr845 and Tyr1101, thereby modulating receptor function and enhancing EGFR signaling [155,156]. Also, beyond canonical ligand-dependent EGFR activation, G protein-coupled receptor (GPCR)-mediated transactivation of EGFR activates Src-dependent signaling (STAT3-, PI3K/AKT-, and MAPK-associated pathways), highlighting the integration between GPCR, EGFR, and Src kinase pathways [157,158,159,160,161,162,163]. Furthermore, Src-dependent signaling can influence necroptotic execution through regulation of MLKL activation, and inhibition of Src using saracatinib, a Src/Abl kinase inhibitor, suppresses MLKL phosphorylation and limits necroptotic execution, indicating that Src-dependent signaling represents a potential regulatory node linking oncogenic kinase signaling with necroptotic regulation [164].

TNFR1 serves as a pivotal signaling hub that coordinates cellular survival and cell death [38]. Upon TNF-α binding, TNFR1 recruits RIPK1, TRADD, TRAF2/5, cIAP1/2, and LUBAC to assemble membrane-associated Complex I [38,122,123]. K63-linked and linear polyubiquitination of RIPK1 within Complex I functions as a molecular docking scaffold that promotes pro-survival NF-κB signaling while suppressing cell death induction [122,123,128]. However, upon the loss of cIAP activity or CYLD-mediated deubiquitination, RIPK1 dissociates from the receptor-associated complex and transitions into cytosolic death-inducing complexes [90,165]. Under conditions of impaired caspase-8 activity, RIPK1 interacts with RIPK3 to assemble the necrosome, thereby shifting TNFR1 signaling from a membrane-associated pro-survival platform to a cytosolic signaling complex that executes necroptosis [18,39].

**Figure 2:**
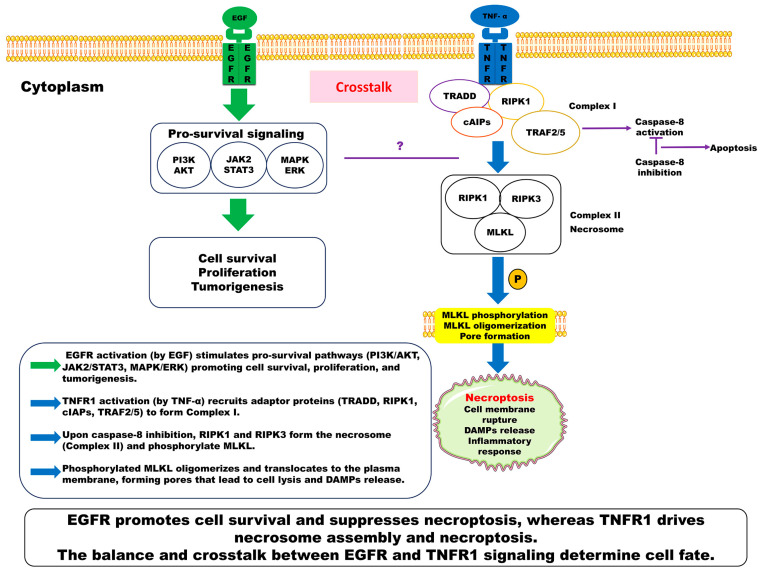
**Crosstalk Between EGFR and TNFR1 signaling in Necroptosis.** EGF-induced EGFR activation promotes cell survival and proliferation through pro-survival pathways, including PI3K/AKT, JAK2/STAT3, and MAPK/ERK. In the context of EGFR- and TNFR1-mediated regulation of necroptosis, EGFR-associated survival signaling influences cellular susceptibility to necroptosis, whereas TNFR1 serves as a major upstream receptor initiating necroptotic signaling through necrosome formation. The potential involvement of EGFR-associated Src signaling in MLKL regulation is indicated by a question mark. The figure was drafted through Servier Medical Art, revised through the free version of ChatGPT (GPT-5.5), and then completed through Servier Medical Art.

Beyond its canonical signaling functions, bidirectional crosstalk between EGFR and TNFR1 has also been reported. TNF-α signaling through TNFR1 inhibits ligand-induced EGFR activation via a TNFR1-dependent mechanism by promoting p38 MAPK-mediated EGFR internalization, thereby attenuating EGFR phosphorylation and downstream signaling. This inhibitory effect requires the TNFR1 death domain and is not mediated by TNFR2 [166]. By contrast, EGFR-dependent phosphorylation of TNFR1 interferes with the early recruitment of RIPK1, thereby suppressing TNFR1-mediated death signaling and potentially limiting necroptotic signaling [53]. Overall, these findings support the concept that bidirectional crosstalk between EGFR and TNFR1 influences tumor cell survival, necroptotic susceptibility, and therapeutic responses in multiple malignancies (Figure 2).

## 5. The Double-Edged Sword of Necroptosis

Necroptosis, a programmed form of lytic cell death regulated by the RIPK1–RIPK3–MLKL signaling axis, is a critical host defense mechanism against intracellular pathogens. However, its uncontrolled activation is recognized as a potent driver of chronic inflammation and autoimmune diseases. By releasing DAMPs and amplifying inflammatory cytokine signaling, necroptosis functionally links innate immune activation to adaptive immune responses. Elucidating the molecular and immunological nuances of this pathway is therefore essential for the development of next-generation therapeutic strategies that selectively modulate necroptotic signaling without compromising systemic immune integrity [28,107].

### 5.1. Immunogenic Tumor Suppression

**Figure 3:**
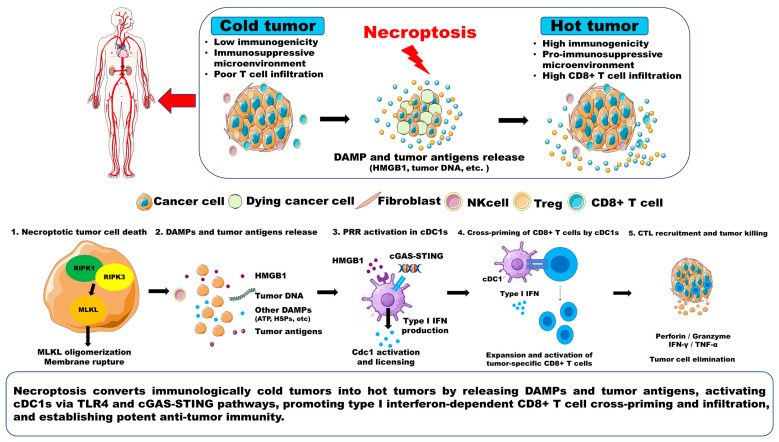
**Conversion of immunologically cold tumors into hot tumors through necroptosis.** Necroptosis remodels the tumor immune microenvironment, thereby reactivating anti-tumor immune responses and promoting the transition of cold tumors into hot tumors. The figure was drafted through Servier Medical Art, revised through the free version of ChatGPT (GPT-5.5), and then completed through Servier Medical Art.

Necroptosis serves as a potent anti-tumor strategy by circumventing intrinsic resistance to cell death. Unlike the immunologically silent characteristic of apoptosis, necroptotic lysis facilitates the release of DAMPs and tumor-derived antigens. In particular, DAMPs released from necroptotic cells, including HMGB1 and tumor-derived genomic DNA, activate pattern recognition receptors, notably TLR4 and the cGAS–STING signaling pathway in type 1 conventional dendritic cells (cDC1s). This signaling axis promotes type I interferon production and licenses cDC1s for efficient CD8+ T-cell cross-priming, thereby linking necroptotic cell death to adaptive anti-tumor immunity. This lethal modality fundamentally remodels the immunosuppressive tumor microenvironment, converting non-immunogenic cold tumors into highly immunogenic hot tumors by activating dendritic cells and recruiting CTLs, thereby reengaging the adaptive immune response against malignant cells (Figure 3) [11,86,167,168,169,170]. Grounded in these mechanistic insights, translational efforts have increasingly shifted from passively observing necroptosis toward therapeutically engineering necroptotic signaling within the TME to overcome immune tolerance. One emerging strategy involves the intratumoral delivery of constitutively active RIPK3 constructs using adeno-associated viral vectors or tumor-targeted mRNA lipid nanoparticles, thereby inducing localized immunogenic necroptotic cell death and promoting the release of tumor-associated antigens and inflammatory mediators. In parallel, growing preclinical evidence suggests that necroptotic induction sensitizes immune-checkpoint blockade-resistant tumors to anti-tumor immunity. For instance, pharmacological targeting of epigenetic silencers, including DNMTs, has been explored as a strategy to restore RIPK3 expression in advanced carcinomas, enhancing responsiveness to anti-PD-1/PD-L1 and anti-CTLA-4 therapies. Mechanistically, necroptosis-associated inflammatory signaling promotes the production of T-cell-recruiting chemokines, such as C-X-C motif chemokine ligands 9 and 10 (CXCL9 and CXCL10), thereby facilitating cytotoxic lymphocyte infiltration into otherwise immunologically cold tumors. Therefore, these findings position therapeutically induced necroptosis as a promising platform for next-generation cancer immunotherapy by functionally coupling tumor cell destruction to adaptive immune activation [11,86,102,169,171,172,173].

### 5.2. Tumor-Promoting Inflammation

**Figure 4:**
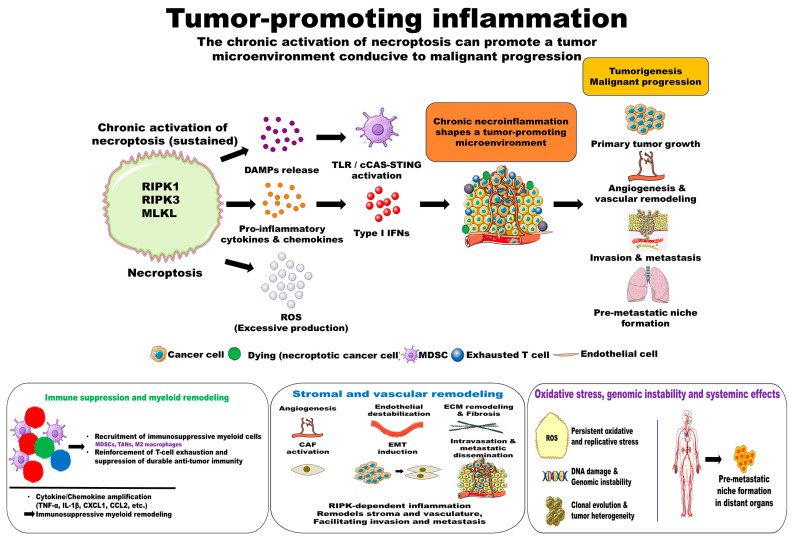
**Tumorigenesis modulation through chronic activation.** Persistent inflammatory signaling enhances the tumor-supportive microenvironment by immune suppression and myeloid and stromal remodeling, along with persistent oxidative stress and genomic instability. The figure was drafted through Servier Medical Art, revised through the free version of ChatGPT (GPT-5.5), and then completed through Servier Medical Art.

Chronic activation of necroptosis can promote a tumor microenvironment conducive to malignant progression, underscoring the paradoxical nature of necroinflammatory signaling. First, a persistent release of intracellular DAMPs and inflammatory mediators establishes a chronic inflammatory milieu that selectively recruits myeloid-derived suppressor cells, tumor-associated neutrophils, and M2-polarized macrophages, thereby reinforcing T-cell exhaustion and suppressing durable anti-tumor immunity. In parallel, sustained RIPK1–RIPK3 signaling amplifies the secretion of pro-inflammatory cytokines and chemokines, including TNF-α, IL-1β, CXCL1, and C-C motif chemokine ligand 2 (CCL2), further driving immunosuppressive myeloid remodeling within the tumor ecosystem. Second, necroptotic signaling directly reshapes the tumor’s stromal and vascular architecture. Chronic activation of RIPK-dependent inflammatory pathways promotes angiogenesis, endothelial destabilization, extracellular matrix remodeling, and epithelial–mesenchymal transition, facilitating tumor cell intravasation and metastatic dissemination. Moreover, inflammatory mediators released during necroptosis activate cancer-associated fibroblasts, generating fibrotic stromal niches that mechanically and biochemically support invasive tumor behavior. Third, sustained necroinflammation promotes persistent oxidative and replicative stress, leading to excessive ROS accumulation, genomic instability, and collateral DNA damage within surrounding stromal and non-malignant tissues. Over time, these processes accelerate tumor heterogeneity, clonal evolution, and the establishment of permissive pre-metastatic niches in distant organs through systemic inflammatory conditioning. Collectively, these findings underscore the double-edged nature of necroptosis, which can function not only as a mechanism of tumor destruction but also as a chronic inflammatory driver of immune dysfunction, stromal adaptation, and metastatic progression (Figure 4).

### 5.3. Systemic Dysregulation: Collateral Tissue Damage

The transition from localized necroptosis to systemic dysregulation can lead to profound pathological risks. Central to this process is the onset of SIRS, which involves an uncontrolled efflux of DAMPs, notably HMGB1. As a result, a hyper-inflammatory cytokine storm occurs. The massive systemic release of TNF, IL-6, and IL-1 leads to microvascular collapse and multi-organ failure. Conversely, chronic exposure of the immune system to sequestered self-antigens can occur when peripheral tolerance breaks down, predisposing the host to autoimmune diseases. Additionally, high EGFR expression levels in sensitive tissues–such as the liver and skin–can sensitize healthy cells to TNF-induced necroptosis, causing severe tissue cell death and impaired organ function [174,175].

## 6. Metabolic Reprogramming and Necroptotic Signaling

Emerging evidence indicates that necroptosis is closely integrated with cellular metabolic programs [27]. Beyond its canonical role as a regulated form of cell death mediated by RIPK1, RIPK3, and MLKL, accumulating evidence indicates a close functional interplay between necroptotic signaling and cellular bioenergetic status. Several studies have demonstrated that RIPK3 directly regulates metabolic enzymes involved in glycogen utilization, glutamine metabolism, and mitochondrial metabolism, suggesting that metabolic remodeling constitutes an integral component of necroptotic signaling rather than a secondary consequence of cell death [11,27,176,177].

Mechanistically, RIPK3 has been shown to phosphorylate and activate multiple metabolic enzymes, including glycogen phosphorylase (PYGL), glutamate-ammonia ligase (GLUL), and glutamate dehydrogenase 1 (GLUD1), thereby promoting metabolic fluxes associated with reactive oxygen species (ROS) generation. Although ROS are not universally required for necroptosis, numerous experimental studies have demonstrated that oxidative stress can amplify necroptotic signaling in a cell type-dependent manner, suggesting that metabolic regulation influences the extent of necroptotic responses [27,178].

The contribution of mitochondria to necroptosis remains an area of active investigation. While MLKL-mediated plasma membrane disruption can proceed independently of classical mitochondrial apoptotic pathways, mitochondrial metabolic activity, redox homeostasis, and cellular energetic state modulate the susceptibility of cells to necroptotic stimulation. These observations suggest that mitochondrial metabolic activity modulates necroptotic susceptibility in certain cellular contexts, although mitochondria are not universally required for necroptotic execution [18,19,28,93,179,180].

Within the TME, fluctuations in oxygen availability, nutrient supply, and cellular metabolic activity influence necroptotic responsiveness. Emerging evidence suggests that metabolic adaptation can modify the cellular context in which RIPK1–RIPK3–MLKL signaling occurs; however, the precise molecular mechanisms linking tumor metabolic states to necroptotic activation remain incompletely defined. Therefore, further investigation is required to establish whether specific metabolic vulnerabilities within the tumor microenvironment can be therapeutically exploited to enhance necroptosis-based anti-cancer strategies [27,177,181].

## 7. Conclusions and Perspectives

In cancer research, increasing attention has been directed toward exploiting necroptosis as a therapeutically actionable form of inflammatory cell death. In this review, we highlight the molecular interplay between the EGFR and TNFR signaling pathways in regulating necroptotic susceptibility and tumor cell fate. EGFR signaling promotes tumor growth, survival, and metastatic progression, in part, by suppressing TNFR-mediated necroptosis via PI3K/AKT- and MAPK-dependent stabilization of pro-survival signaling networks and inhibition of necrosome activation. The EGFR–TNFR signaling axis is a promising therapeutic framework for selectively modulating necroptotic competence in cancer cells. However, effective therapeutic exploitation of necroptosis will require precise control over the balance between tumoricidal efficacy and collateral inflammatory toxicity. First, modulating key signaling nodes, including RIPK1 and caspase-8, can redirect cell death programs toward necroptosis in apoptosis-refractory malignancies. Second, epigenetic restoration of RIPK3 expression through DNA methyltransferase or histone deacetylase inhibition can re-sensitize tumor cells to necroptotic signaling and enhance responsiveness to conventional cytotoxic therapies. Third, integrating necroptosis-inducing strategies with immunotherapy potentiates anti-tumor immunity through the release of DAMPs and tumor-associated antigens, thereby improving responses to immune checkpoint blockade or adoptive cellular therapies. Concurrently, emerging nanoparticle-based delivery systems and future gene-editing approaches enable spatially restricted activation of RIPK3–MLKL signaling within tumor tissues while minimizing systemic inflammatory injury.

Collectively, these findings support the concept that controlled induction of tumor-selective necroptosis represents a promising avenue for next-generation oncological interventions. Future therapeutic strategies will likely combine necroptosis modulation with immunotherapy, metabolic targeting, and locoregional delivery platforms to maximize anti-tumor efficacy while limiting pathological necroinflammation.

## Data Availability

No new data were created or analyzed in this study. Data sharing does not apply to this article.
